# Neck Pain in Fibromyalgia: Treatment with Exercise and Mesotherapy

**DOI:** 10.3390/biomedicines11030892

**Published:** 2023-03-14

**Authors:** Dalila Scaturro, Fabio Vitagliani, Gabriele Signa, Sofia Tomasello, Luigi Giuseppe Tumminelli, Alessandro Picelli, Nicola Smania, Giulia Letizia Mauro

**Affiliations:** 1 Department of Surgical, Oncological and Stomatological Disciplines, University of Palermo, 90127 Palermo, Italy; 2 Faculty of Medicine and Surgery, University of Catania, 90121 Catania, Italy; 3 Neuromotor and Cognitive Rehabilitation Research Center, Physical and Rehabilitation Medicine Section, Department of Neurosciences, Biomedicine and Movement Sciences, University of Verona, 37100 Verona, Italy; 4 Neurorehabilitation Unit, Department of Neurosciences, University Hospital of Verona, 37100 Verona, Italy

**Keywords:** mesotherapy, rehabilitation, fibromyalgia, neck pain, chronic musculoskeletal pain

## Abstract

Background and Objectives: Fibromyalgia is a very common musculoskeletal disease. The purpose of this study is to assess, on a population of fibromyalgic patients, the clinical efficacy of antalgic mesotherapy with diclofenac and thiocolchicoside in the treatment of cervical pain reduction for improvement of the functional capacity and quality of life of these patients. Materials and Methods: We conducted an observational study of 78 fibromyalgia patients recruited using our hospital database. Based on the different types of treatment received, the patients were divided into two groups: the treatment group (TG), who received antalgic mesotherapy with diclofenac, thiococolchicoside, and mepivacaina; and the placebo group (PG), who received mesotherapy with sodium chloride solution. Patients in both groups also received the same rehabilitation protocol of 20 sessions. The primary outcome evaluated was the extent of pain. The secondary outcomes were the functional capacity and quality of life. Results: Pain improved both in the treatment group (7.4 ± 1.2 vs. 5.1 ± 1.1; *p* < 0.05) and placebo group (7.5 ± 1.4 vs. 6.1 ± 1.6; *p* < 0.05). The treatment group, compared to the placebo group, also showed significant statistical improvements in functional capacity (NDI: 35.6 ± 5.23 vs. 19.3 ± 3.41; *p* < 0.05) and quality of life (SF-12: 18.3 ± 4.11 vs. 33.1 ± 2.41; *p* < 0.05). Conclusions: Mesotherapy treatment with diclofenac and thiocolchicoside is a safe and effective procedure in the management of neck pain in fibromyalgia patients in the short term in terms of pain reduction, functional recovery and quality of life.

## 1. Introduction

Fibromyalgia is a musculoskeletal disease of unknown etiology, which affects, on average, 2.10% of the world population; it affects 2.31% of the European population [1,2]. It is the second most common rheumatological disorder, after osteoarthritis, with a higher prevalence between 20 and 50 years of age [3]. It is more common among women and has significant repercussions on work activities and the socio-emotional status [4,5].

It has been hypothesized that fibromyalgia is of a multifactorial origin, with an underlying clear genetic predisposition [6]. Possible causes may include alterations in the release of neurotransmitters, hypersensitivity of the central nervous system, alterations in the hypothalamic–pituitary axis, alterations in the release of pro-inflammatory cytokines and impaired balance between oxidizing and anti-oxidizing substances [1,6].

The immune system and neuroinflammation therefore play a crucial role in CNS sensitization, including the activation of nociceptors. Many studies [7,8,9,10] in the literature have addressed and analyzed the different possible mechanisms regarding the pathogenesis of fibromyalgia; in fact, it is associated with disorders in the processing of pain by the central nervous system (CNS), such as hyperalgesia and allodynia, characteristic symptoms of fibromyalgic syndrome. Both imply a central sensitization, namely greater processing of the CNS of painful stimuli, which develops as a result of the plasticity of neuronal synapses.

The stimulation of nociceptors is triggered by proinflammatory mediators such as adenosine triphosphate (ATP) and interleukin-1β (IL-1β), resulting in increased sensitivity to pain and contributing to the symptomatology of FM.

Recent studies have also shown that purinergic signaling is often associated with pain and inflammatory responses [11]. Indeed, the purine molecule ATP has been shown to play an important role in regulating nociceptive transmission in different regions of the spinal cord and brain and promotes the release of mediators involving pain. Therefore, targeting ATP-specific receptors (e.g., P2X and P2Y) could be effective in modulating pain conditions [12,13].

The main clinical feature of fibromyalgia is the presence of widespread chronic pain, similar, for clinical presentation, physiopathology, and neuropharmacology, to neuropathic pain. Muscles, skin, ligaments, and tendons are affected, often accompanied by allodynia and hyperalgesia, as well as asthenia, morning stiffness, sleep disturbances, paresthesia, headache, anxiety and depression, irritable bowel syndrome, Raynaud’s phenomenon, and menstrual pain [2,5].

Chronic pain in these patients is often localized in the cervical spine region, making it responsible for acute and protracted episodes of cervical pain, associated with concomitant states of muscle tension of the suboccipital muscles, trapezius muscle, and shoulder blade lift muscle. This is often associated with impairment in neck movement [14,15,16].

Fibromyalgia is a very often underestimated and neglected condition, but over time, it strongly affects the quality of life and physical and working capacity of affected individuals [17].

In patients with fibromyalgia, cervical pain is a very common and highly disabling multifactorial symptom, a concerning problem for modern society that is often responsible for limiting social and work activities, and although it is not the most common musculoskeletal disorder, it appears to play a certain role in the worsening of the patient’s psychological–physical–social condition, also representing an economic burden that is not to be underestimated, given both the costs of processing and any problems related to reduced working and functional productivity [18]. 

Current treatment of fibromyalgia is based on a multidisciplinary and gradual approach: lifestyle and behavior changes, pharmacological treatments (e.g., antidepressants, muscle relaxants, and anticonvulsants), and non-pharmacological treatments [19]. The latter includes various interventions such as aerobic exercises, flexibility exercises, strength training, stretching and body-awareness therapies (e.g., Feldenkrais therapy), and psychotherapy [20,21,22,23]. All of these treatments are essentially aimed at symptom management and quality of life improvement [24].

Exercise is the most appropriate non-pharmacological treatment, but the heterogeneity of the disease must be individualized according to the physical function of the patients, the severity of the pain, and other symptoms [25]. It can be performed in combination with relaxing massage therapy, connective massage, and electrotherapy. It has benefits in terms of improving pain, anxiety and depression, health, and quality of life [14,26].

Antalgic mesotherapy, an increasingly emerging approach, can be considered a valid therapeutic alternative in fibromyalgic patients [27]. Mesotherapy is defined as the use of intra- or subcutaneous injections containing liquid mixtures of compounds (drugs, plant extracts, vitamins, and other ingredients) for the treatment of loco-regional conditions [27].

The great advantage offered by mesotherapy consists in the amount of drug used, minimal compared to the dosage required in the conventional ways of administration; additionally, therapeutic action is “targeted”, which means that it acts in the localized painful part of the body [27,28]. When combined with physical and manual therapies, antalgic mesotherapy accelerates pain remission and functional recovery [27].

The pharmacological combination most used for antalgic mesotherapy is the combination of NSAIDs, muscle relaxants, and local anesthetics [27,28,29]. This association exploits the combination of an anti-inflammatory and decontracting action, which has an important antalgic effect [29]. 

Only a few studies have currently evaluated the efficacy of mesotherapy in the management of cervicalgia [29], while its effectiveness is long documented in the literature for chronic lumbar pain [30,31], where it can be considered as a viable treatment option for pain and disability reduction [31,32].

The purpose of this study is to assess, on a population of fibromyalgic patients, the clinical efficacy of antalgic mesotherapy with diclofenac and thiocolchicoside in the treatment of fibromyalgia for cervical pain reduction and the improvement of the functional capacity and quality of life of these patients.

## 2. Materials and Methods

### 2.1. Study Design

We conducted a case–control observational study at the U.O.C. of Recovery and Functional Rehabilitation of the A’O.’U. Paolo Giaccone of Palermo on patients diagnosed with neck pain suffering from fibromyalgia. The study was conducted according to the ethical guidelines of the Declaration of Helsinki; the local ethics committee “Palermo 1” approved the study, with reference number 03/2022; information and data were handled according to Good Clinical Practice (GCP) guidelines. All participants signed an informed consent form at enrollment to collect clinical data. Using our hospital database, we included a consecutive series of fibromyalgia patients.

### 2.2. Setting

A total of 78 fibromyalgia patients met the inclusion criteria and were included in our study. Of these, 39 patients had undergone analgesic mesotherapy treatment with a combination of diclofenac fl 50 mg/2 mL, thiococolchicoside fl 4 mg/2 mL, and mepivacaine fl 10 mg/1 mL and were assigned to a treatment group (TG), while the remaining 39 patients underwent mesotherapy treatment using a placebo consisting of 9 mL of sodium chloride solution and were assigned to a placebo group (PG). The mesotherapy sessions were carried out by the same physiatrist doctor with many years of experience (D.S.) every week for a total of 7 sessions. Additionally, the patients of both groups received the same rehabilitation protocol of 20 sessions, under the supervision of a physiotherapist with many years of experience.

### 2.3. Participants

The following inclusion criteria were used: age 18–55 years; clinical diagnosis of fibromyalgia according to the 2016 revised American College of Rheumatology (ACR) criteria [24]; clinical objectivity of neck pain not associated with neurological and/or vascular deficits; absence of septic processes in place; and written informed consent for study participation. Patients with allergic reactions to NSAIDs or with neoplastic diseases and patients previously treated with analgesic mesotherapy were excluded. A patient met the 2016 ACR criteria for fibromyalgia if the following 3 conditions were met: (1) Widespread Pain Index (WPI) ≥ 7 and Symptom Severity Scale (SSS) score ≥ 5 OR WPI of 4–6 and SSS score ≥ 9. (2) There must be generalized pain, defined as pain in at least 4 of 5 regions; pain in the jaw, chest, and abdomen are not included in the definition of generalized pain. (3) Symptoms must have generally been present for at least 3 months [33].

### 2.4. Clinical Evaluation

Demographic (age, gender, BMI) and clinical (comorbidity and presence of muscle tension) information was taken into consideration. The following rating scales were also administered: Numeric Rating Scales (NRS) [34], to assess the extent of pain; the Neck Disability Index (NDI) [35], to assess the patient’s cervical spine function; the Fibromyalgia Impact Questionnaire (FIQ) [36], to evaluate the impact of the disease on these patients; and a 12-Item Short Form Survey (SF-12) [37], to evaluate the patient’s quality of life considering their pathological condition. These scales were performed at the first mesotherapy session (T0) and 2 months after the end of the last mesotherapy session (T1) by the same physiatrist (D.S.). The primary outcome measure was the extent of pain, using the NRS scale. The secondary outcomes evaluated were the functional capacity of the patient, using the NDI and FIQ scales; and quality of life, using the SF-12 scale. 

The NRS scale is a quantitative assessment scale whereby patients are asked to evaluate their pain on a defined scale, from 0 to 10, best reflecting the intensity of pain at that specific time [34]. 

The Neck Disability Index (NDI) is a self-administered questionnaire, i.e., completed by patients with cervical pain, who assess how neck pain affects their daily life activities. The NDI consists of 10 sections, each of which investigates how pain affects different aspects of daily life (intensity of pain; personal care; lifting weights; reading; headaches; focusing; working; driving; sleeping; recreation). Each section contains 6 possible answers with the score varying from 0 to 5, where 0 corresponds to no difficulty or pain while 5 corresponds to inability to perform the activity or disabling pain. The complex score varies between 0 and 50, where higher scores are associated with increased disability [35]. 

The Fibromyalgia Impact Questionnaire (FIQ) is a questionnaire consisting of 10 questions, divided into three parts. The first part contains 11 items related to the ability in the last week to carry out activities of daily life, with a score varying between 0 (always) and 3 (never). In the second and third parts, the number of days of the last week in which the patient has felt well and has not been able to carry out their work (including housework) due to the symptoms of fibromyalgia is required. Questions 4 to 10 are related to the extent of fibromyalgia interference with one’s work, the intensity of pain and asthenia, the quality of night rest, the intensity of rigidity, and the presence of anxiety or depression; the answers vary from 0 (no disturbance) to 10 (very important disturbance), marked on a horizontal linear scale. The maximum FIQ score, corresponding to the highest degree of disability, is 100; in patients with fibromyalgia, the mean FIQ values are around 50, while only patients with severe clinical pictures have results above 70 [36].

The SF-12 scale is a shortened version of the Short Form 36 Items Health Survey (SF-32) questionnaire and is a generic indicator of the patient’s quality of life and biopsychosocial well-being. Through 12 of the 36 questions of the original questionnaire, this scale investigates 8 aspects related to the state of health: physical activity, role limitations due to physical health, emotional state, physical pain, perception of the general state of health, vitality, social activities, and mental health. The synthesis of the scores totaled allows 2 synthetic indices to be built: a physical health index (PCS-12) and a mental health index (MCS-12). The lower the score of the two indices, the higher the level of disability [37].

### 2.5. Mesotherapy Procedure

The mesotherapy was performed by an experienced and trained physician using a 10 mL syringe on which 4 mm and 6 mm 30 Gauge sterile disposable needles were mounted (Mesorelle, Biotekne SRL, Casalecchio di Reno, Italy). At each treatment session, a pharmacological mixture containing 2 mL (50 mg) of diclofenac, 2 mL (2 mg) of thiocolchicoside, and 1 mL (10 mg) of mepivacaine was prepared. Then, 4 mL of sodium chloride solution was added. 

The operator starts with the disinfection of the skin with gauze soaked in chlorhexidine. After the application, the disinfectant was left to work for 5 min.

A measure of 0.1–0.2 cc of this pharmacological mixture was administered at each injection site injected at a depth of 1–3 mm, with a distance between two injection points of about 2 cm and without causing papules. At the end of the injections, disinfection of the skin was performed again, a covering patch was applied, and the patient remained in observation for about 10 min, noting the possible appearance of adverse reactions. Patients were advised not to wet the injection area within the next 12 h [38]. 

### 2.6. Rehabilitation Protocol

The rehabilitation sessions were held three times a week with a duration of 100 min and under the supervision of an experienced physiotherapist. Each session consisted of 40 min of aerobic exercises, 30 min of CO_2_ laser, and 15 min of transcutaneous electric nerve stimulation (TENS) on specific tender points.

Aerobic exercises promote body weight loss and help reduce the work of antigravity muscles, with muscle relaxant and analgesic effects. Aerobic exercises consisted of three parts. The first part of the 10-minute warm-up consists of breathing and stretching exercises performed at 70–80% of the maximum heart rate; patients performed segmental active muscle stretching without the assistance of the therapist. Large muscles were chosen for their role in muscle chains of the overall postural re-education method. The triceps surae, buttocks, ischiotibials, paravertebrals, latissimus dorsi, hip adductor, and pectoral were targeted. The intensity of the elongation was gradually increased to the point of moderate discomfort and the position was maintained for 30 s, according to the recommendation of the American College of Sports Medicine [39]. The second part of the 20-minute duration consisted of progressive strengthening exercises, proprioceptive exercises, and neuromuscular coordination exercises. The equipment used included dumbbells (upper limbs) and shin guards (lower limbs). In the first two sessions, no load was used. Subsequently, 0.5 kg was added every week according to patient tolerance. Patients were instructed to perform a series of eight repetitions of endurance exercises for the following muscles: triceps surae, quadriceps, adductors and abductors of the hip, hip flexors, flexors, elbow extensors, major pectorals and rhomboids [36].

The low-energy laser is a widely used tool used in the treatment of musculoskeletal disorders, including fibromyalgia. It was applied to the three most painful points. It acts through an anti-inflammatory, anti-edema, and analgesic action, with short- and long-term efficacy in the treatment of fibromyalgia [40].

TENS is a non-invasive method with analgesic action that is carried out through the excitation of the sensory nerves and the stimulation of the gate pain mechanism and/or the opioid system. It allows a reduction in pain and fatigue, improving hyperalgesia and the functional capacity in patients with fibromyalgia [40].

### 2.7. Statistical Analysis

The data obtained were indexed on an Excel sheet and analyzed with the statistical software R. The sample size was calculated with the formula below:

$$n=\frac{z_{\alpha /2}^{2}\sigma^{2}}{\varepsilon^{2}}\to n=\frac{z_{\alpha /2}^{2}-\pi (1-\pi )}{\varepsilon^{2}}$$

The type I error is equal to 0.01 (the quantile in the formula is equal to 2.58). The denominator *ε* = 0.05 is the maximum error acceptable to the researcher, and the choice of *ε* is arbitrary. Finally, we use the worst-case scenario of the proportion equal to 0.5. The formula gave a result equal to 69.99. Consequently, the number of 78 patients considered was sufficient to prove our thesis.

The descriptive analysis was performed based on the mean and standard deviation. For the statistical test, we used three different tests: the *t*-test to compare means for quantitative variables, Mood’s median test to compare medians for ordinal variables, and the test for two proportions. The results were considered statistically significant with a *p*-value < 0.05.

## 3. Results

Table 1 shows the general characteristics at baseline of the patients recruited (Table 1). The average age was 49.3 ± 7.21 years, with 91.1% (*n* = 71) identifying as female and the remaining 8.9% (*n* = 7) identifying as male.

A total of 58% of patients had at least one comorbidity, among which the most common were: anxiety–depressive syndrome (24%), insomnia (21%), and headache (16%). 

A total of 87% (*n* = 68) had states of tension on the trapezius, paravertebral, shoulder blade lift, and rhomboid muscles. In 38% of patients, these muscular tensions were present bilaterally, while in the remaining 62%, they were monolateral. A total of 73% of patients had an important limitation of the ROM of the cervical spine, with a greater involvement of the movement of flexion and left and right rotation. 

The mean value of the NRS scale was 7.3 ± 2.13, with more than 50% of patients reporting a worsening of symptoms during the night. Paresthesia in the neck and/or upper limbs was present in 33% of patients. The average score on the NDI scale was 36.2 ± 5.3, with the worst scores being highlighted for work, ability to concentrate, drive, and sleep. Finally, the average score of the FIQ scale was 55.9 ± 6.1, while the SF-12 questionnaire was 17.4 ± 8.25, with worse scores affecting the physical health. No statistically significant difference was observed between the two basal groups, except for a greater presence of comorbidity in the placebo group (*p* < 0.05).

Patients showed 100% treatment compliance. Placebo patients showed no adverse reactions. Five patients in the treatment group showed adverse reactions, especially erythema and itching. These adverse reactions were sporadic and individual and occurred in two consecutive sessions in only two cases. 

**Figure 1:**
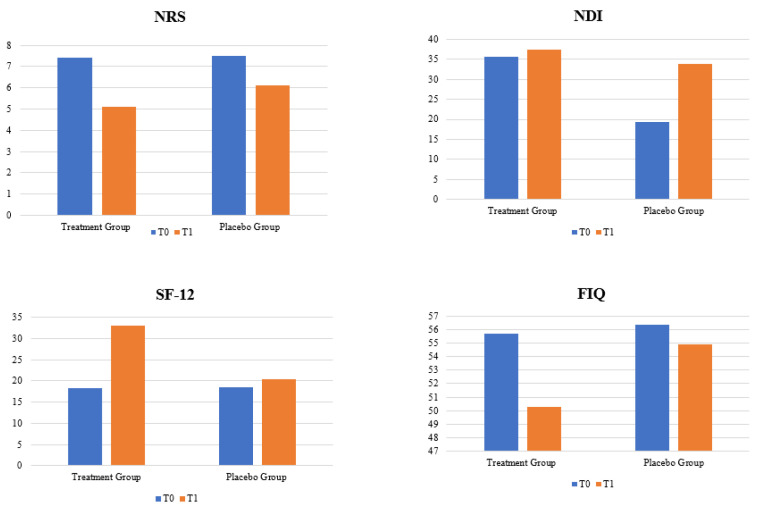
Variation in primary and secondary outcomes in treatment group and placebo group at T1.

Table 2 and Figure 1 show the effects of different mesotherapeutic treatments in patients at T1 (Table 2). Regarding pain, both groups at the end of mesotherapy sessions showed significant improvement. In the treatment group, pain was reduced by 2.3 points according to the NRS scale (7.4 ± 1.2 vs. 5.1 ± 1.1; *p* < 0.05), while in the placebo group at T1, pain was reduced by about 1.4 points according to the NRS scale (7.5 ± 1.4 vs. 6.1 ± 1.6; *p* < 0.05).

The treatment group also showed statistically significant improvements at T1 in the NDI scale score (35.6 ± 5.23 vs. 19.3 ± 3.41; *p* < 0.05) and the SF-12 scale (18.3 ± 4.11 vs. 33.1 ± 2.41; *p* < 0.05). No statistically significant improvement was observed in the placebo group for the NDI scale (37.4 ± 5.4 vs. 33.8 ± 4.8; *p* = 0.11) and SF-12 (18.6 ± 3.8 vs. 20.3 ± 4.8; *p* = 0.09).

No statistically significant improvement was observed for the FIQ score, either in the treatment group (55.7 ± 7.7 vs. 50.3 ± 4.7; *p* = 0.09) or in the placebo group (56.4 ± 5.8 vs. 54.9 ± 4.5; *p* = 0.20).

## 4. Discussion

In this study, we compared the action/effectiveness of mesotherapy with the usage of conventional drugs (NSAIDs—muscle relaxants) in combination with a rehabilitation protocol compared to mesotherapy with only a placebo in combination with a rehabilitation protocol in fibromyalgic patients with cervicalgia in terms of pain reduction, disability improvement, and quality of life. 

The results obtained at the two-month revaluation show a statistically significant improvement in pain reduction for both the control group and treatment group.

Regarding the treatment group, this result could partly derive as a direct consequence of the reduction in muscle tension states induced by drugs; on the other hand, the beneficial effects of mesotherapy with associations of NSAIDs and muscle relaxants are now known in the literature [31,41]. This kind of association has also shown positive results in the treatment of other diseases. Iklerd Akbas et al. [42] investigated the role of intradermal mesotherapy with NSAIDs, thiocolchicoside, and lidocaine in the treatment of LBP, resulting in a better clinical result (reduction in pain) than using systemic drugs. Their results suggest that mesotherapy is more effective in pain relief; this effect begins earlier and continues longer than with the systemic administration of NSAIDs. The need for analgesic use at any time within 24 h after therapy was statistically lower in the mesotherapy group. 

Other authors [43] have studied the comparison between a mesotherapy with normal saline solution or with a cocktail of drugs in patients suffering from cervicalgia for spondyloarthrosis. In their work, both groups showed effective short-term results in reducing pain and disability. However, only patients treated with a cocktail of drugs showed improvement three months after treatment.

Ferrara et al. [44] also analyzed the short- and long-term effects of mesotherapy using a mixture of drugs compared to normal saline in the treatment of patients with chronic spinal pain (CSP). At the end of treatment, both groups had improved symptoms, while after 12 weeks of follow-up, the improvement was significantly greater in patients treated with the drug cocktail than in those treated with saline.

Ronconi et al. [31] investigated the effects of diclofenac mesotherapy in patients with chronic lower back pain. From the data obtained, the authors underlined the effectiveness of this treatment in terms of pain reduction and functional disability related to the pathology, proposing this treatment as a possible alternative in the management of these patients. This could be an indirect consequence given by the reflex effect of the penetration of the needle at the level of nociceptive stimuli and/or by the distension of the tissue and the sensitive fibers due to the steric presence of the liquid. In fact, the puncture effect of the needle itself could be a reason for muscle stress, inducing loco-regional relaxation due to the penetration of the needle. The puncture of the needle stimulates skin and subcutaneous receptors (reflex effect), which determines an increase in endorphin levels after the introduction of the needle. This concept is taken up in acupuncture practice [45,46]. Microinjections, without medication, seem to facilitate the rebalancing of the nociceptive system through a series of local and complex actions that are not yet well known involving nociceptive receptors and nociceptive mechanisms centered on feedback mechanisms [47].

Moreover, we must consider how the role of rehabilitation, therapeutic and aerobic physical exercise, is of fundamental importance for the fibromyalgic patient, as widely expressed in the literature, to obtain a reduction in and control of pain and improvement in terms of mobility and joint recovery. In fact, even the control group achieved a significant reduction in pain. Borcher et al. [48] report that an individualized exercise regimen in combination with cognitive–behavioral therapy leads to a reduction in pain and fatigue.

Other authors report how bi- or tri-weekly aerobic exercises and therapeutic exercise focused on muscular strengthening has a positive impact on the reducing pain and also on mood and depression [49] and that exercise brings greater benefits to the fibromyalgic patient than drug treatment alone [50]. 

The improvement of functional indices and quality of life is also statistically significant for the treatment group; an increase, although not statistically significant, turned out to be the overall psycho-physical impact on these patients. 

Few studies in the literature have investigated the application of mesotherapy in the treatment of chronic cervicalgia. Paolucci et al. [51] compared the effectiveness of trigger point treatment in chronic cervicalgia by comparing mesotherapy with lidocaine and “dry” mesotherapy without any substance. The authors showed how meso-therapeutic treatment in both cases brings some benefits, resulting in the superior use of anesthesia, both in terms of pain and in terms of functional gain and quality of life.

In the treatment group, along the same lines as the results of Paolucci et al. [51], we also achieved positive results with regard to disability in fibromyalgic patients with cervicalgia. At the end of the mesotherapy sessions, the patients showed excellent improvements in the ROM of the cervical spine, reductions in muscle tension states associated with cervicalgia, and improvements in the NDI scale score.

Its main advantages are low invasiveness, easy reproducibility and speed of execution, and the possibility of administering the drug directly in the pain site while avoiding all possible complications, both systemic and non-systemic, resulting from the passage of different drugs into the gastrointestinal tract [47]. 

Finally, patients in the treatment group expressed a significant and statistically significant improvement in their quality of life, with increased participation in recreational and social activities.

This could be the result of the efficacy and safety of mesotherapeutic treatment and its low incidence of side effects perceived by the patient himself. Among possible side effects, we can divide complications between infectious and non-infectious complications, the first being more concerning than the latter, which can be avoided by following strict hygiene and sterility regulation. Non-infectious complications mainly include dermatological complications such as the formation of granulomas, microabscesses, and more common local skin reactions, urticarial rashes, and intolerance to the substances [52]. The side effects we experienced were only local skin reactions, which were self-resolving and did not lead patients to abandon treatment. This is a very important aspect because the fibromyalgic patient has an important psychological and behavioral involvement that affects the mood and possible somatization of various disorders. The idea of being able to receive treatment that proves effective without having to resort to systemic active ingredients can help the patient to deal more positively with their pathological condition, with less impact on their psychological sphere.

Mesotherapeutic treatment could therefore be an alternative therapeutic solution that avoids the use of pharmacological principles with systemic action, favoring even more compliance, especially in patients with comorbidity and those fearful of taking medication. The latter category includes fibromyalgic patients, who often resort to abusing drugs with different consequences for the body and economic burdens.

The main strength of our experience is the uniqueness of the project, as we were the first to consider the use of mesotherapy with and without drugs in the management of cervicalgia in fibromyalgic patients. 

However, our experience has some limitations, including the low sample cohort, which does not allow use to generalize the results to all of the effects shown; and the lack of a follow-up. Long-term follow-up could show the real duration of the benefits obtained and the ability to prevent further episodes of neck pain as well as the impossibility to distinguish the real effectiveness of the treatment with respect to the possible placebo effect on the psychological component.

## 5. Conclusions

Considering our limited understanding of the etiopathogenesis of fibromyalgia, which includes a complex multifactoral system involving increased central sensitivity for nociception in which inflammation plays a fundamental role, the innovation of this study lies in the lack of literature exploring similar treatments: the comparison between a medicated intradermal treatment and a placebo for the treatment of cervicalgia in fibromyalgic patients. By acting safely, you work on multiple components of pathology, inflammation, and antalgic contractures. The clinical management of the fibromyalgic patient is still under discussion today, given the considerable mental and psychological components associated with the pathology. Our experience emphasizes the effectiveness and use of a technique such as mesotherapy, in some of its possible uses in the management of cervical pain in fibromyalgic patients. Mesotherapeutic treatment with diclofenac and thiocolchicoside has proven to be a safe and effective procedure in the management of cervicalgia in fibromyalgic patients, and superior, in the short term, to the placebo in terms of pain reduction, functional recovery, and quality of life. Moreover, thanks to its safety profile, it could be considered a first-line therapeutic approach in fibromyalgia. Of course, further studies are still needed to assess their effectiveness over time. Our goal was to create a starting point for further research developments that include conservative and/or minimally invasive treatments, such as mesotherapy, in treating cervical pain and improving the quality of life of these patients.

## Figures and Tables

**Table 1:** General characteristics at baseline.

Characteristics	Total (*n* = 78)	Treatment Group (*n* = 39)	Placebo Group (*n* = 39)	*p*-Value
Age, mean ± SD	49.3 ± 6.2	50.1 ± 4.7	48.7 ± 4.4	0.18
Sex, *n* (%)				0.21
Female	71 (91.1)	34 (87.2)	37 (94.8)	
Male	7 (8.9)	5 (12.8)	2 (5.2)	
BMI (Kg/m^2^), mean ± SD	27.6 ± 3.4	28.3 ± 4.1	27.3 ± 3.8	0.27
Comorbidity, *n* (%)				<0.05
Yes	45 (57.7)	20 (51.3)	25 (64.1)	
No	33 (42.3)	19 (48.7)	14 (35.9)	
Muscle tension, *n* (%)				0.38
Yes	68 (87.2)	35 (89.7)	33 (84.6)	
No	10 (12.8)	4 (10.3)	6 (15.4)	
NRS, mean ± SD	7.3 ± 2.1	7.4 ± 1.2	7.5 ± 1.4	0.73
NDI, mean ± SD	36.2 ± 5.3	35.6 ± 5.2	37.4 ± 5.4	0.14
SF-12 scale, mean ± SD	17.4 ± 8.2	18.3 ± 4.1	18.6 ± 3.8	0.74
FIQ, mean ± SD	55.9 ± 6.1	55.7 ± 7.7	56.4 ± 5.8	0.65

**Table 2:** Primary and secondary outcomes in the treatment group and placebo group at T1.

Characteristics	Treatment Group (*n* = 39)	Placebo Group (*n* = 39)
NRS, mean ± SD		
T0	7.4 ± 1.2	7.5 ± 1.4
T1	5.1 ± 1.11	6.1 ± 1.6
*p*-value	<0.05	<0.05
NDI, mean ± SD		
T0	35.6 ± 5.2	37.4 ± 5.4
T1	19.3 ± 3.4	33.8 ± 4.8
*p*-value	<0.05	0.11
SF-12, mean ± SD		
T0	18.3 ± 4.1	18.6 ± 3.8
T1	33.1 ± 2.4	20.3 ± 4.8
*p*-value	<0.05	0.09
FIQ, mean ± SD		
T0	55.7 ± 7.7	56.4 ± 5.8
T1	50.3 ± 4.7	54.9 ± 4.5
*p*-value	0.09	0.20

## Data Availability

Data used to support the findings of this study are available from the corresponding author upon request.
